# A New Electrochemical Sensor for the Detection of Ketoconazole Using Carbon Paste Electrode Modified with Sheaf-like Ce-BTC MOF Nanostructure and Ionic Liquid

**DOI:** 10.3390/nano13030523

**Published:** 2023-01-28

**Authors:** Somayeh Tajik, Fatemeh Sharifi, Behnaz Aflatoonian, Antonio Di Bartolomeo

**Affiliations:** 1 Research Center of Tropical and Infectious Diseases, Kerman University of Medical Sciences, Kerman P.O. Box 76169-13555, Iran; 2 Department of Physics “E.R. Caianaiello”, University of Salerno, Fisciano, 84084 Salerno, Italy

**Keywords:** sheaf-like Ce-BTC MOF, nanostructure ketoconazole sensing, chemically modified sensors, electrochemical detection

## Abstract

An ultrasensitive and selective voltammetric sensor with an ultratrace-level detection limit is introduced for ketoconazole (KTC) determination in real samples using a modified carbon paste electrode with a sheaf-like Ce-BTC MOF nanostructure and ionic liquid. The as-synthesized nanostructure was characterized by several techniques, including energy-dispersive X-ray spectroscopy (EDX), X-ray diffraction (XRD), and field emission scanning electron microscopy (FE-SEM). The electrocatalytic performance of the developed electrode was observed by cyclic voltammetry (CV), differential pulse voltammetry (DPV), linear sweep voltammetry (LSV), and chronoamperometry. The limit of detection (LOD) of the developed sensor for KTC is 0.04 μM, and the response was found to be in the dynamic concentration range of 0.1–110.0 μM in a phosphate buffer solution. The proposed electrode exhibits acceptable electrocatalytic activity for KTC oxidation with a high sensitivity of 0.1342 μA·μM^−1^. The ability of the fabricated sensor to monitor KTC in real aqueous samples is demonstrated using standard addition data.

## 1. Introduction

**Figure 1:**
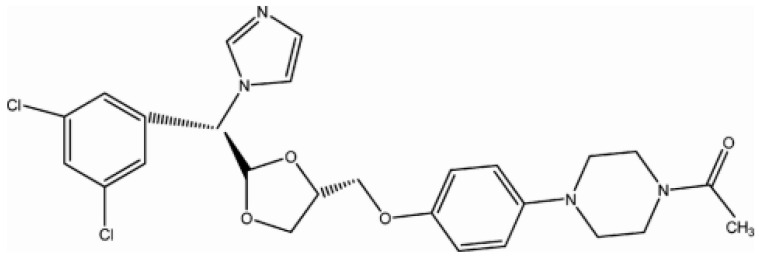
Chemical structure of ketoconazole.

Ketoconazole (KTC) is a synthetic imidazole Cis-1-acetyl-4-[4-[2-(2,4-dichlorophenyl)-2-(1H-imidazole-1-ylmethyl)-1,3-dioxolon-4-yl] methoxy] piperazin (see Figure 1) that acts as an oral antifungal agent with a broad spectrum against systematic and superficial fungi. KTC can block the production of fungal ergo sterol, thereby disrupting the permeability of the cell membrane and ultimately preventing its growth. Exposure to KTC can be associated with changes in cytochrome P-450, which has central functions in the detoxification of biologically active substances as well as in the activity of epoxide hydrolase [1,2,3,4,5,6]. In addition to damaging the fungal mitochondrial and microsomal enzymes, its mechanism of action is the destruction of the fungal cytoplasmic membrane. It is extensively applied in people with immunodeficiency and prostate cancer [7,8]. Compared to other identical agents, it has a wide range of functions and few complications. Some of the applications of KTC are the formulation of products such as shampoo, gel, cream, lotion, and foam [9,10,11].

It is essential to quantify KTC through ultraviolet detection [12,13,14,15], spectrophotometry [16,17], and capillary electrophoresis [18]. Despite various advantages, these methods have some disadvantages because they are costly, time-consuming, and environmentally incompatible, and require much experience. Electrochemical approaches have aroused great attention because of their merits such as cost-effectiveness, simplicity, portability, rapidity, and high selectivity in biological fluids, cosmetics, and pharmaceutical products [19,20,21,22,23,24,25,26].

An interesting and recent approach is chemically modified electrodes (CMEs), which are mainly used for selective and sensitive electrochemical determination. They can mediate the electrode reaction through a significant reduction in over-potential, independent of pH, with a fast and stable reaction in redox response [27,28,29,30,31,32,33,34,35,36,37]. In 1979, an electrochemical approach called the chemical modification of the carbon paste electrode surface was proposed [38,39]. Researchers have introduced various modifiers to enhance the electrocatalytic activity of various carbon-supported electrodes, some of which include metal nanoparticles (NPs), metal oxide NPs, conductive polymers, and metal–organic frameworks (MOFs) [40,41,42,43,44,45].

MOFs are porous materials with infinite crystal networks that are the result of metal ion bonding and function as coordination centers possessing multifunctional organic molecules. MOFs are of particular importance due to their beneficial properties, such as controllability, adjustable capabilities, high porosity, large specific surface area, host–guest interplay, and great thermal stability. Various applications have been reported for MOFs, including gas storage/separation, catalysis, drug delivery, and detection with electrochemical applications, which can be attributed to the electrochemical performance of metal ions and the ordered porous framework. Various types of MOFs are synthesized at high temperature, high pressure, or with organic solvents, which not only increases the cost of fabrication but also makes large-scale production difficult [46,47,48,49,50,51,52,53]. The synthesis of mixed-valence Ce-MOFs occurs rapidly at an ambient temperature in aqueous solution, saving considerable time and energy costs [54,55]. In addition, these compounds can catalyze reversible redox responses with enzymatic-like performance, thus having a good catalytic impact on KTC oxidation.

Certain properties have been reported for ionic liquids (ILs) such as toxicity, chemical–thermal stability, high ionic conductivity, and the ability to dissolve various organic and inorganic substances [56]. There is a broad field of application of room temperature ionic liquids (RTILs) in analytical chemistry. RTILs are mixed with carbon substances to construct the conductive composites applied in electrochemical structures due to advantages such as a low vapor pressure, high conductivity, a wide electrochemical window, satisfactory chemical–thermal stability, and negligible toxicity [57,58,59,60,61,62].

In the present study, for KTC detection, the ionic liquid exhibited an excellent sensitivity and electric conductivity with highly rapid electron transfer in electroanalysis, and the sheaf-like (SL) Ce-BTC MOF nanostructure (NS) enhanced the electrochemical profile of CPE. For the first time, the as-fabricated modified electrode possessed high KTC sensing activity with a broad linear dynamic range, ultralow detection limits and outstanding sensitivity.

## 2. Experimental Section

### 2.1. Devices and Materials

An Autolab galvanostat/potentiostat device (PGSTAT 302N, Metrohm Ltd., Herisau, Switzerland) equipped with GPES 4.9 software was utilized to perform all electrochemical determinations. The routine three-cell system applied in this study consisted of a modified CPE (IL/SL Ce-BTC MOF NS/CPE) working electrode, a Metrohm Ag/AgCl/KClsat reference electrode and a platinum wire auxiliary electrode. The pH of fresh solutions prepared with deionized water (Direct-Q^®^ 8 UV water purification system; Millipore; Germany) was adjusted with a digital pH meter (Metrohm 713, Switzerland). An X-ray diffractometer (Cu/Kα radiation, λ = 1.5418 nm; XRD; Panalytical X’Pert Pro; the Netherlands) was employed to record the XRD patterns. A scanning electron microscope (MIRA3, Tescan, Czech Republic) connected to an EDX system was used to perform FE-SEM analysis.

In our study, all chemicals were of analytical grade from Sigma-Aldrich with no need for additional purification. H_3_PO_4_ was used for the preparation of phosphate buffer solution. The pH adjustment was performed with NaOH.

### 2.2. Fabrication of SL Ce-BTC MOF NS

The method proposed by Liu and colleagues was followed to construct the SL Ce-BTC MOF NS [63]. Thus, 0.2171 g of Ce(NO_3_)_3_·6H_2_O was dissolved in 1 mL of deionized water to prepare metal salt aqueous solution with a pH value of 3.5. Then, the solution was slowly poured into 1,3,5 benzene tricarboxylic acid (1,3,5-BTC; 0.10 g) water–ethanol solution (1:1 *v/v*; 40 mL) at an ambient temperature while vigorously stirring for 10 min. Next, the centrifugation was carried out to obtain the white precipitate that formed, followed by rinsing thoroughly with ethanol/deionized water and drying.

### 2.3. Fabrication of Electrodes

The Ce-BTC MOF NS and ionic-liquid-modified carbon paste electrode (Ce-BTC MOF/IL/CPE) was fabricated by mixing 0.8 mL of IL, 0.4 g of Ce-BTC MOF NS, and 0.9 g of paraffin oil and graphite powder in a mortar to obtain a uniform wet paste, which was then compressed in the bottom of a glass tube. A copper wire was embedded in the glass tube behind the mixture to achieve an electrical contact. After removing the excess paste, a weighing paper was applied to polish and create a new surface. The comparison was performed for CPE; IL/CPE, unmodified (bare) CPE, Ce-BTC MOF/CPE, and IL-CPE.

### 2.4. Preparation of Real Specimens

Five KTC tablets (containing 200 mg of KTC) were first powdered, and then 200 mg of the powder was dissolved in 25 mL water under ultrasonication. Next, variable volumes of as-diluted solution was diluted to the mark of a 25 mL volumetric flask with PBS (pH 7.0). The standard addition method was followed to determine the KTC content.

The instantly refrigerated urine specimens, at a certain volume (10 mm), were centrifuged at 2000 rpm for 20 min. Then, the supernatant was filtered by a filter, and various volumes of it were diluted to the mark of a 25 mL volumetric flask with PBS (pH 7.0). Next, the diluted specimens were spiked with various concentrations of KTC.

## 3. Results and Discussion

### 3.1. Characterization of SL Ce-BTC MOF NS

**Figure 2:**
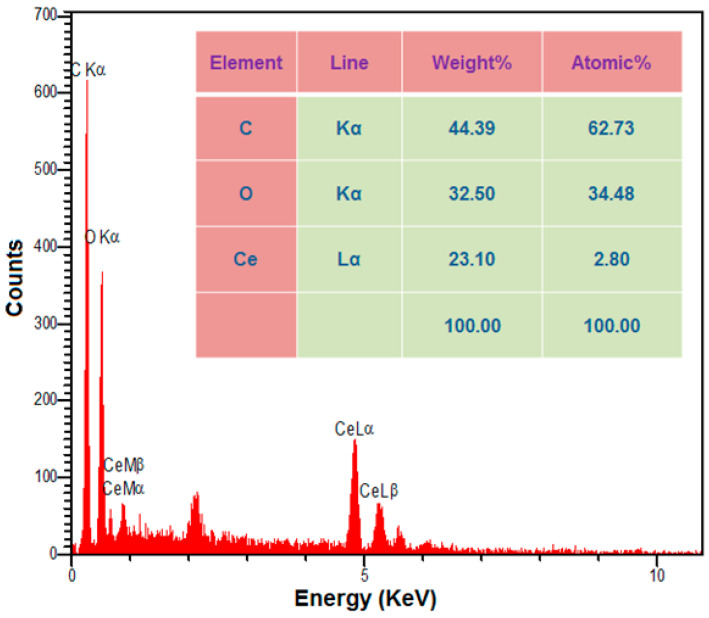
EDX spectrum of sheaf-like Ce-BTC MOF.

The EDX analysis was employed to examine the SL Ce-BTC MOF NSs’ chemical composition, the results of which revealed signals related to Ce, C, and O that verified the fabrication of Ce-BTC MOF (see Figure 2).

**Figure 3:**
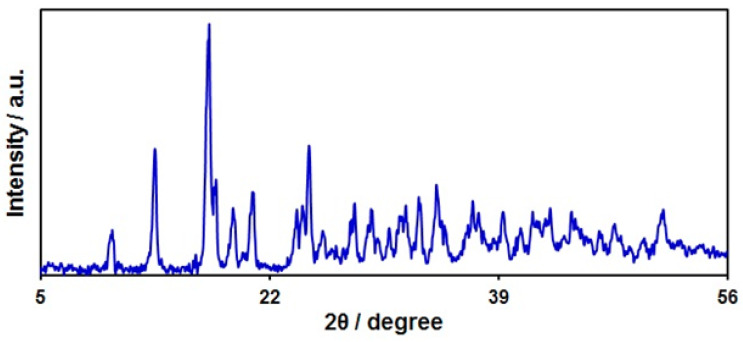
XRD pattern of sheaf-like Ce-BTC MOF.

The crystal structure of SL Ce-BTC MOF NS was determined by the XRD pattern (Figure 3). Some sharp peaks appeared in the XRD pattern of Ce-BTC MOF, which ranged from 5° to 50°, confirming a good crystal structure of the as-fabricated sensor. The characteristic peaks at Ce-MOF were in line with a previous study on Ce-BTC MOF [63].

**Figure 4:**
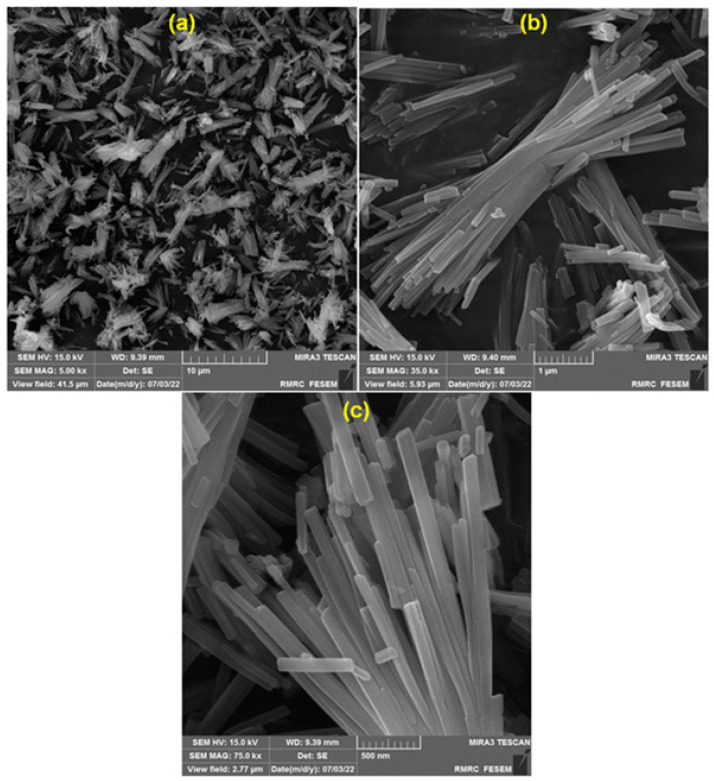
FE-SEM images of sheaf-like Ce-BTC MOF nanostructures at different magnifications (**a**) 5′000, (**b**) 35′000, and (**c**) 75′000.

Figure 4a–c illustrate the FE-SEM images used to examine the Ce-BTC MOF’s morphological and structural features. It can be observed that the Ce-BTC MOF consists of an array of dispersed nanorods (with a mean diameter of ~90 nm) that are joined together in the middle to construct straw-SL structures.

### 3.2. Electrochemical Determinations

The electrocatalysis of KTC on the surface of Ce-BTC MOF/IL/CPE could be significantly influenced by the pH of the supporting electrolyte. The influence of pH on the determination of KTC (50.0 μM) in the exposure to PBS was examined on the surface of the modified electrode at variable pH values between 2.0 and 9.0. The pH value of 7.0 had the greatest oxidation peak current of KTC. Hence, pH = 7.0 was chosen as the best value for the next test.

**Figure 5:**
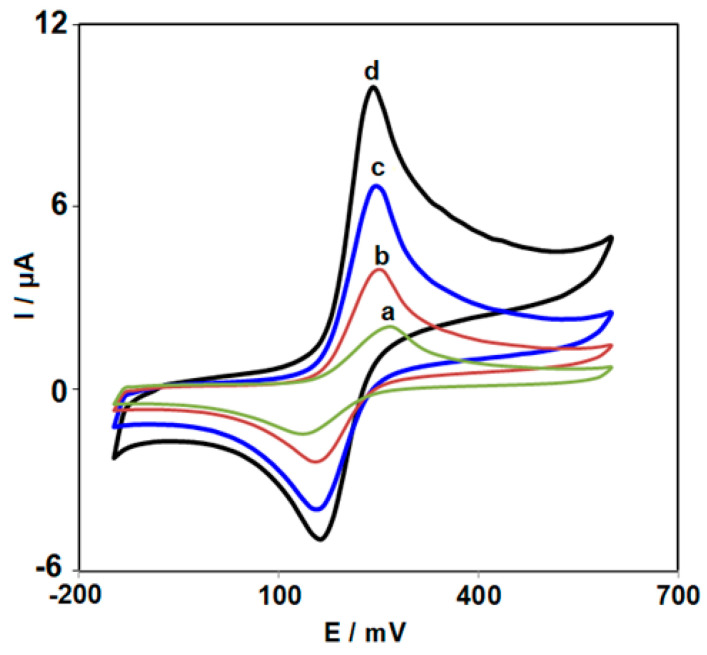
Cyclic voltammograms captured for oxidation of KTC (70.0 µM) in PBS (0.1 M; pH = 7.0) on: (**a**) unmodified CPE; (**b**) Ce-BTC MOF/CPE; (**c**) IL/CPE; and (**d**) Ce-BTC MOF/IL/CPE, with a scan rate of 50 mV/s.

To compare with unmodified CPE, IL-CPE, and Ce-BTC MOF/CPE, cyclic voltammetry was performed to explore the electrochemical behavior of Ce-BTC MOF/IL/CPE in relation to KTC (70.0 μM) in 0.1 M PBS at 50 mV/s. The cyclic voltammograms (CVs) were captured for all as-prepared electrodes in 0.1 M PBS with the pH value of 7.0 and a scan rate of 50 mV/s. Figure 5 shows a strong peak current resulting from the electrode surface modification with the porous and conductive MOF NS. The ionic liquid could facilitate the electron transfer, thus remarkably boosting the oxidation peak current. The I_pa_ value (μA) was estimated at 1.8 for CPE, 3.9 for Ce-BTC MOF/CPE, 6.5 for IL-CPE, and 9.9 for Ce-BTC MOF/IL/CPE in relation to the KTC oxidation. These results demonstrated the success of the method for CPE modification in the proposed electrode fabrication.

### 3.3. The Role of Scan Rate (ʋ) in the Experiments

**Figure 6:**
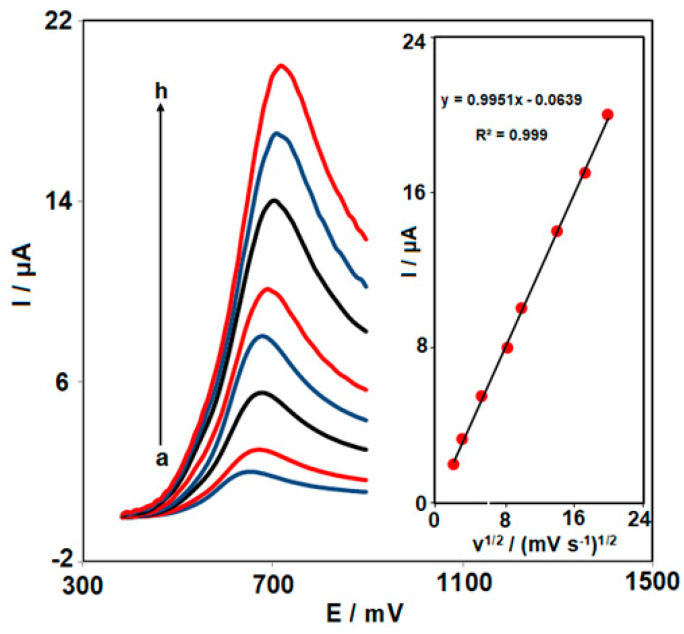
LSVs captured for the oxidation of KTC (50.0 µM) on the Ce-BTC MOF/IL/CPE under variable scan rates (a–h: 5, 10, 30, 70, 100, 200, and 400 mV/s). Inset: the correlation of I_pa_ with v^1/2^.

Linear sweep voltammetry was performed to determine the scan rate influence on the electrochemical performance of KTC on the surface of Ce-BTC MOF/IL/CPE. According to the results (Figure 6), there was a gradual elevation in the oxidation peak current when the scan rate increased to 400 from 5 mV/s. As seen in Figure 6 (inset), there was linearity for the anodic peak currents (I_pa_) vs. the scan rate square root (v^1/2^), R^2^ = 0.999. A linear elevation occurred for the peak current of oxidation with the v^1/2^, highlighting a diffusion-controlled process for the KTC electro-oxidation on the Ce-BTC MOF/IL/CPE surface in the tested range of scan rates.

**Figure 7:**
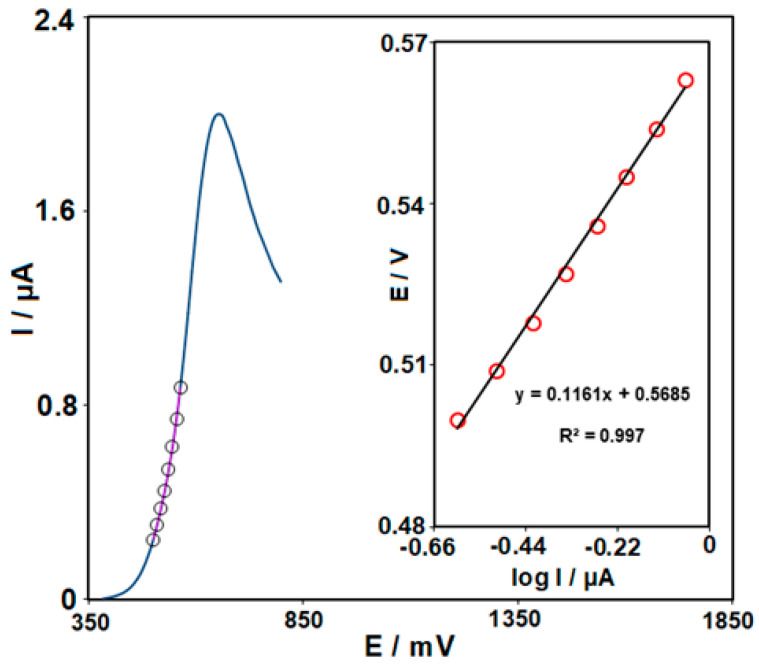
LSV (at 5 mV s^−1^) of the electrode in 0.1 M PBS (pH = 7.0) containing 50.0 μM KTC. The points are the data used in the Tafel plot (inset).

To analyze the rate-determining step, a Tafel plot was obtained in accordance with the points of LSV for KTC (50.0 μM) on the surface of Ce-BTC MOF/IL/CPE (scan rate = 5 mV/s). As seen in Figure 7 (inset), the Tafel plot was applied to compute the electron transfer coefficient (α = 0.49) with the slope of 2.3RT/n (1 − α) F (0.1161 V).

### 3.4. Chronoamperometric Exploration

**Figure 8:**
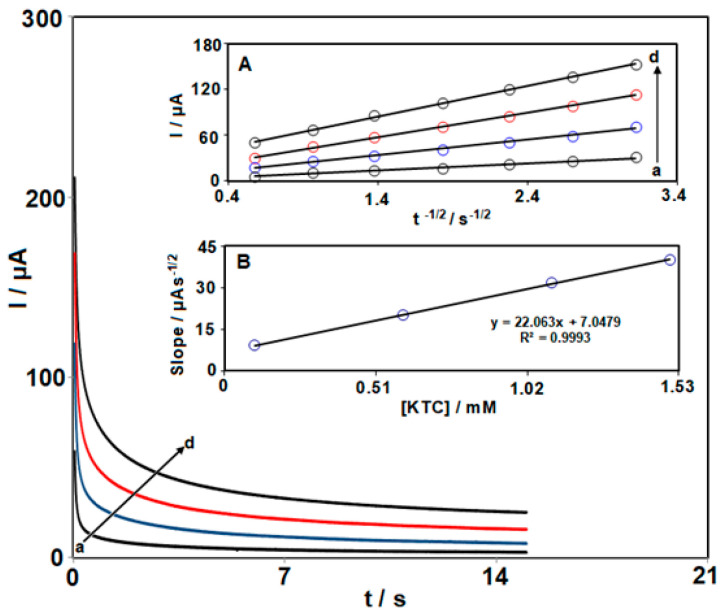
Chronoamperometric behavior of Ce-BTC MOF/IL/CPE in PBS (0.1 M; pH = 7.0) at a potential of 300 mV for variable KTC contents (a–d: 0.1, 0.6, 1.1, and 1.5 mM). Insets: (**A**) plots of I vs. t^−1/2^; (**B**) plots of the slopes from the straight lines vs. KTC level.

Chronoamperometric analysis was carried out for the KTC determination on the surface of Ce-BTC MOF/IL/CPE (Figure 8) at the electrode potential of 0.3 V against variable KTC levels (100.0, 600.0, 1100.0 and 1500.0 μM) with a buffer solution pH of 7.0 (Figure 8). Cottrell’s equation (I = nFAD^1/2^C_b_π^−1/2^t^−1/2^) was run to describe the current of the electrochemical reaction under the limited condition of mass transport for the electroactive KTC with the diffusion coefficient of D [64]. Figure 8A illustrates the plots of I against t^−1/2^ used with the best fit for variable KTC levels. The slopes from the straight lines were diagramed versus variable KTC levels (Figure 8B). Based on the Cottrell equation and slope, the D was found to be 5 × 10^−6^ cm^2^/s.

### 3.5. Standard Curve, Linear Dynamic Range, and Limit of Detection

**Figure 9:**
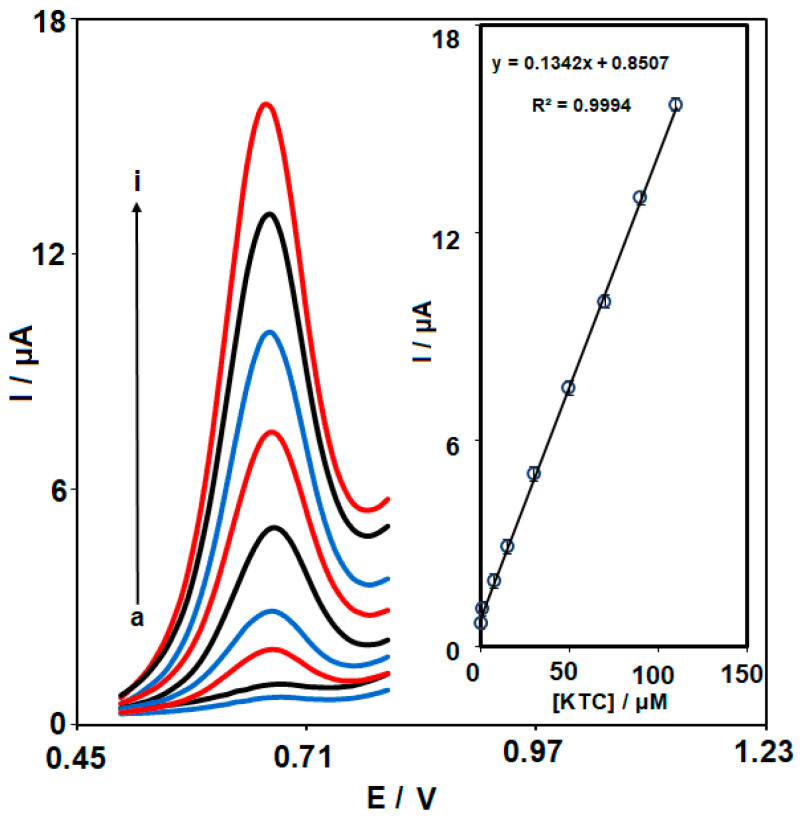
DPVs captured for the oxidation of variable KTC contents on the Ce-BTC MOF/IL/CPE under variable contents (a–i: 0.1, 1.0, 7.5, 15.0, 30.0, 50.0, 70.0, 90.0, and 110.0 µM). Inset: calibration curve of voltammetric response (I_pa_) against KTC level.

The limit of detection and linear dynamic range were computed by the differential pulse voltammograms (DPVs). Figure 9 depicts the DPVs from Ce-BTC MOF/IL/CPE at variable KTC levels in 0.1 M PBS. The oxidation peak current of KTC was gradually boosted when the KTC concentration increased, verifying the high potency of the as-fabricated electrochemical sensor in the electro-oxidation of KTC. The changes in the peak currents of KTC oxidation on the Ce-BTC MOF/IL/CPE surface vs. the KTC content are presented in Figure 9 (inset). The linear dynamic range was as wide as 0.1–110.0 μM. Moreover, the limit of detection, C_m_, of KTC was calculated using the following equation:

C_m_ = 3S_b_/m

where m is the slope of the calibration plot (0.1342 μA/μM), and S_b_ is the standard deviation of the blank response which was obtained from 15 replicate measurements of the blank solution. The limit of detection obtained using this method was 0.04 μM.

A comparison of the electroanalytical parameters for KTC detection with those previously reported in the literature [8,65,66,67,68] is presented in Table 1.

### 3.6. Interference Study

The effect of several interference species on the determination of KTC was studied. The results show that the interfering effects of Zn^2+^, Fe^3+^, Cu^2+^, K^+^, Na^+^, Co^2+^, and Mg^2+^ ions, and glycerin, ascorbic acid, lactose, glucose, and sodium benzoate on the anodic peak current of KTC is less than 5%. Hence, the Ce-BTC MOF/IL/CPE sensor has a superior selectivity for KTC.

### 3.7. Real Sample Testing

The practical applicability of the as-fabricated modified electrode was analyzed by sensing KTC in pharmaceuticals and urine specimens in accordance with the standard addition method. Table 2 shows the obtained experimental data. The recovery rates were recorded between 96.7% and 103.6%, and the relative standard deviations were ˂3.5%, which show the potential of the as-fabricated electrode for sensing the KTC in the real matrices.

## 4. Conclusions

In this study, for the first time, we modified the surface of the carbon paste electrode with the Ce-BTC MOF NS and ionic liquid for electrochemically sensing KTC. The as-developed electrode had high sensitivity and selectivity in detecting the study analyte, with a narrow limit of detection. The linear dynamic range was as broad as 0.1–110.0 μM (R^2^ = 0.9906), and the limit of detection was as narrow as 0.04 μM in the optimized setting. Further, the diffusion coefficient was 5 × 10^−6^ cm^2^/s, and the electron transfer coefficient was 0.49. The practical applicability of the as-fabricated modified electrode was confirmed by sensing KTC in real pharmaceuticals and urine specimens, with satisfactory recoveries.

## Figures and Tables

**Table 1:** Comparing the electroanalytical performance of the Ce-BTC MOF/IL/CPE with other electrochemical sensors for KTC determination.

Electrochemical Sensor	Method	Linear Range	Limit of Detection	Ref.
Gold nanoparticles/carbon paste electrode	Square wave voltammetry curves	1.0–80.0 μM	0.1 μM	[8]
Multi-walled carbon nanotubes/glassy carbon electrode	Differential pulse voltammetry	1.0–30.0 μM	0.44 μM	[65]
Gold nanoparticle/glassy carbon electrode	Cyclic voltammetry	20.0–100.0 μM	2.3 μM	[66]
Beta-cyclo-dextrin/glassy carbon electrode	Differential pulse voltammetry	1.0–8.0 × 10^−5^ M	10.54 × 10^−8^ M	[67]
Nitrogen-doped carbon/glassy carbon electrode	Cyclic voltammetric	47.0–752.0 μM	3.0 μM	[68]
Ce-BTC MOF/IL/CPE	Differential pulse voltammetry	0.1–110.0 μM	0.04 μM	This Work

**Table 2:** Voltammetric sensing of KTC in real specimens using Ce-BTC MOF/IL/CPE (n = 5).

Sample	Spiked (μM)	Found (μM)	Recovery (%)	R.S.D. (%)
Tablet	0	3.5	-	3.2
	1.0	4.4	97.8	1.9
	2.0	5.7	103.6	2.8
	3.0	6.6	101.5	2.2
	4.0	7.4	98.7	2.5
Urine	0	-	-	-
	5.0	5.1	102.0	1.9
	6.0	5.8	96.7	3.5
	7.0	7.1	101.4	2.1
	8.0	7.9	98.7	2.3

## Data Availability

The data presented in this study are available on request from the corresponding authors.
